# A Randomized, Blinded, Vehicle-Controlled Dose-Ranging Study to Evaluate and Characterize Remdesivir Efficacy Against Ebola Virus in Rhesus Macaques

**DOI:** 10.3390/v16121934

**Published:** 2024-12-18

**Authors:** Elizabeth E. Zumbrun, Carly B. Garvey, Jay B. Wells, Ginger C. Lynn, Sean A. Van Tongeren, Jesse T. Steffens, Kelly S. Wetzel, Darrell L. Wetzel, Heather L. Esham, Nicole L. Garza, Eric D. Lee, Jennifer L. Scruggs, Franco D. Rossi, Elizabeth S. Brown, Jessica M. Weidner, Laura M. Gomba, Kristan A. O’Brien, Alexandra N. Jay, Xiankun Zeng, Kristen S. Akers, Paul A. Kallgren, Ethan Englund, J. Matthew Meinig, Jeffrey R. Kugelman, Joshua L. Moore, Holly A. Bloomfield, Sarah L. Norris, Tameka Bryan, Christie H. Scheuerell, Jesse Walters, Nevena Mollova, Christiana Blair, Darius Babusis, Tomas Cihlar, Danielle P. Porter, Bali Singh, Charlotte Hedskog, Sina Bavari, Travis K. Warren, Roy Bannister

**Affiliations:** 1 United States Army Medical Research Institute of Infectious Diseases, Frederick, MD 21702, USA; carly.b.garvey.ctr@health.mil (C.B.G.); jay.b.wells.ctr@health.mil (J.B.W.); ginger.c.lynn.ctr@health.mil (G.C.L.); sean.a.vantongeren.ctr@health.mil (S.A.V.T.); jtsisme@gmail.com (J.T.S.); kelly.s.wetzel2.ctr@health.mil (K.S.W.); darrell.l.wetzel.ctr@health.mil (D.L.W.); heather.l.esham.civ@health.mil (H.L.E.); eric.d.lee2@gmail.com (E.D.L.); jscruggs@easternvetpath.com (J.L.S.); franco.d.rossi.civ@health.mil (F.D.R.); elizabeth.s.brown34.ctr@health.mil (E.S.B.); jessica.m.weidner@gmail.com (J.M.W.); laura.gomba@nih.gov (L.M.G.); kristan.a.obrien.ctr@health.mil (K.A.O.); alexandra.n.jay.civ@health.mil (A.N.J.); xiankun.zeng.civ@health.mil (X.Z.); kristen.akers@nih.gov (K.S.A.); paul.a.kallgren.ctr@health.mil (P.A.K.); ethan.englund.phd@gmail.com (E.E.); james.m.meinig.civ@health.mil (J.M.M.); jeffrey.r.kugelman.mil@health.mil (J.R.K.); joshmoore49@gmail.com (J.L.M.); holly.a.bloomfield.civ@health.mil (H.A.B.); sarah.l.norris2.civ@health.mil (S.L.N.); sina.bavari@tonixpharma.com (S.B.); travis.warren@nih.gov (T.K.W.); 2 Geneva Foundation, Tacoma, WA 98402, USA; 3 PharPoint Research, Inc., Wilmington, NC 28401, USA; tameka.bryan@pharpoint.com; 4 Labcorp Early Development Laboratories, Madison, WI 53704, USA; christie.scheuerell@labcorp.com (C.H.S.); jesse.walters@labcorp.com (J.W.); 5 Gilead Sciences, Inc., Foster City, CA 94404, USA; nevena.mollova@gilead.com (N.M.); chris.blair@gilead.com (C.B.); darius.babusis@gilead.com (D.B.); tomas.cihlar@gilead.com (T.C.); danielle.porter@gilead.com (D.P.P.); bali.singh@gilead.com (B.S.); charlotte.hedskog@gilead.com (C.H.); roy.bannister@gilead.com (R.B.)

**Keywords:** Ebola virus, filovirus, remdesivir, RDV, GS-5734, Veklury, nonhuman primate, rhesus macaque

## Abstract

Ebola virus (EBOV) causes severe disease in humans, with mortality as high as 90%. The small-molecule antiviral drug remdesivir (RDV) has demonstrated a survival benefit in EBOV-exposed rhesus macaques. Here, we characterize the efficacy of multiple intravenous RDV dosing regimens on survival of rhesus macaques 42 days after intramuscular EBOV exposure. Thirty rhesus macaques underwent surgical implantation of telemetry devices for the fine-scale monitoring of body temperature and activity, as well as central venous catheters, to enable treatment administration and blood collection. Treatment, consisting of a loading dose of RDV followed by once-daily maintenance doses for 11 days, was initiated 4 days after virus exposure when all animals were exhibiting disease signs consistent with incipient EBOV disease as well as quantifiable levels of EBOV RNA in plasma. In the RDV treatment groups receiving loading/maintenance doses of 5/2.5 mg/kg, 10/5 mg/kg, and 20/10 mg/kg, a total of 6 of 8 (75%), 7 of 8 (87.5%), and 5 of 7 (71.4%) animals survived, respectively. In the vehicle control group, one of seven animals (14.3%) survived. The improved survival rate compared to the control group was statistically significant only for the 10/5 mg/kg RDV treatment group. This treatment regimen also resulted in a significantly lower systemic viral load compared to the vehicle control after a single RDV treatment. All three RDV regimens produced a significantly lower systemic viral load after two treatments. For most animals, RDV treatment, regardless of dose, resulted in the amelioration of many of the clinical–pathological changes associated with EBOV disease in this model.

## 1. Introduction

Members of the genus *Orthoebolavirus* have caused more than 30 outbreaks in sub-Saharan Africa since their discovery in 1976. *Orthoebolavirus zairense* (EBOV; Ebola virus) has been responsible for more than two-thirds of these outbreaks [1], including the largest outbreak to date in 2013–2016, in which a total of 28,646 cases resulted in 11,323 fatalities in West Africa [2]. Across outbreaks, the average case–fatality rate has been around 50% but has reached as high as 90%, according to the World Health Organization [3]. Outbreaks can start with at least one zoonotic transmission (spillover) event or after reactivation from a human survivor of a past outbreak; subsequent human-to-human spread occurs via contact with infected tissues, body fluids, or contaminated fomites [4,5,6].

The small-molecule antiviral remdesivir (RDV; GS-5734) is a monophosphoramidate prodrug of an adenosine nucleoside analog with antiviral activity against numerous RNA viruses, including EBOV [7,8]. Inside target cells, RDV is converted to the active triphosphate metabolite that inhibits the filovirus RNA-dependent RNA polymerase. In rhesus macaques exposed intramuscularly (IM) to EBOV, Warren et al. found that once-daily RDV, administered intravenously (IV) beginning 3 days after challenge, was fully protective. Among 12 animals receiving 10 mg/kg RDV—whether as daily 10 mg/kg doses for 12 days or as a single 10 mg/kg loading dose followed by 11 days at 3 mg/kg—all 12 (100%) survived, whereas all control animals succumbed to infection. Treated animals also exhibited significant decreases in plasma viral RNA compared to control animals [8]. The therapeutic efficacy of RDV has also been demonstrated in rhesus macaques exposed to aerosolized EBOV and those exposed to Marburg virus (MARV) or Sudan virus (SUDV) by the IM route [9,10,11].

RDV was evaluated, along with three other drugs (mAb114, REGN-EB3, and ZMapp), in a randomized clinical trial during the 2018 EBOV outbreak in the Democratic Republic of the Congo [12]. Although RDV showed some clinical benefit, it did not perform as well as the mAb114 and REGN-EB3 arms of the trial. Nevertheless, RDV remains promising as an EBOV countermeasure—whether as a standalone therapy or for use in combination with monoclonal antibody therapeutics. In rhesus macaques infected with MARV or SUDV by the IM route, combining RDV with a monoclonal antibody therapeutic increased overall survival with a late treatment initiation time of up to 6 days after challenge [9,13]. In addition, RDV may have enhanced penetrance into immune-privileged tissues, thereby potentially reducing viral persistence. This idea is supported by the results of the PREVAIL IV clinical trial, which demonstrated that RDV reduced the presence of EBOV RNA in the semen of EBOV disease (EVD) survivors [14].

The study reported herein relies on a disease model characterized through a natural history study of IM EBOV, conducted under Good Laboratory Practices (GLPs) [15]. In the IM rhesus EBOV model as described by Warren et al., disease manifestations, such as fever, viral RNA in blood, inflammation, lymphocytolysis, and reduced activity, are all present in most animals by Day 4 post-inoculation (PI) [15]. Here, we have chosen Day 4 PI as the treatment initiation time based on findings from the natural history study showing the occurrence of multiple disease manifestations on Day 4 PI.

In this study, we characterized the effect of multiple IV dosing regimens of RDV on survival 42 days after IM EBOV exposure in rhesus macaques. Additionally, we (a) characterized the effect of IV RDV regimens on viral load, disease-related clinical signs, temperature and activity, and alterations in hematology, coagulation, and serum chemistry parameters; (b) assessed the effect of RDV treatment on anatomic pathology findings in postmortem analyses; (c) evaluated the pharmacokinetic (PK) parameters of RDV and relevant metabolites in plasma of EBOV-infected rhesus macaques following once-daily administrations via IV infusion; and (d) assessed the viral genomic sequence in RDV-treated EBOV-exposed animals.

## 2. Materials and Methods

### 2.1. Quality System

This study was conducted in accordance with GLPs to align with regulatory guidance set forth by the U.S. Food and Drug Administration in the U.S. Code of Federal Regulations Title 21, Part 58.

### 2.2. Experimental Design

Due to the number of animals involved in this study and the complexity of the procedures, the study was conducted in two independent iterations. For each iteration, 16 animals were randomly assigned to four treatment groups (*n* = 4 per group per iteration). One animal in each iteration was removed prior to the onset of treatment administration due to catheter damage. Specifically, 1 animal in Group 1 (vehicle) was removed from the study on Day 3 PI, and 1 animal in Group 4 (20/10 mg/kg RDV) was removed from the study on Day 4 PI prior to the first dose of the test article. Data from these animals are not included here. After removing the 2 animals that experienced catheter patency issues, a total of 30 rhesus macaques (15 males and 15 females) were subjected to experimental procedures (Table 1).

On Day 0, each animal was exposed to a target dose of 1000 plaque-forming units (pfu) EBOV/Kikwit. The calculated challenge dose was 399.5 pfu and 331.5 pfu in Iterations 1 and 2, respectively, based on plaque assay of challenge material. Injections were administered via the IM route in the right quadriceps in a volume of 0.5 mL.

RDV was administered by 30 min IV infusion via a femoral central venous catheter (CVC) once daily to three groups of animals beginning 4 days after virus exposure and continuing for a total of 12 days. Specifically, on Day 4 PI, animals received an initial loading dose of 5, 10, or 20 mg/kg RDV; on Days 5–15 PI, once-daily maintenance doses of 2.5, 5, or 10 mg/kg RDV, respectively, were administered, survival permitting. A control group was included as a comparator study arm and was administered matching vehicle once daily beginning on Day 4 PI and continuing for a total of 12 days. The weight of each animal was obtained on the day of virus inoculation (Day 0); these weights were used for dose–volume determination for all administered doses of RDV or vehicle.

### 2.3. Animals, Husbandry, Randomization, and Blinding

Experimentally naive Chinese-origin rhesus macaques (*Macaca mulatta*) were obtained for this study from an AAALAC-accredited domestic breeding colony. Animals had no preexisting antibodies to EBOV glycoprotein and tested negative for herpes B, simian immunodeficiency virus, simian T-lymphotropic virus, simian retrovirus, *Trypanosoma cruzi*, tuberculosis, *Salmonella*, and *Shigella*. Animals with a history of gastrointestinal disorders or any disease or injury requiring treatment within 30 days prior to study initiation were excluded from the study. Each animal underwent surgical implantation of DSI M00 telemetry devices (35–54 days prior to challenge) and two CVCs for jugular and femoral vein access (20–24 days prior to challenge). Surgeries were conducted as described previously [15], with the exception that a femoral catheter was also placed. Animals were transferred for acclimation to the animal biosafety level 4 (ABSL-4) laboratory 8 days prior to inoculation. At the time of EBOV exposure, animals weighed 4.1–8.8 kg and were 4–7 years of age.

For each iteration, animals were randomly assigned to treatment groups, stratified by sex and balanced by body weight (Table 1). Exceptions to the randomization are as follows. The study director assigned 1 Group 1 animal to Group 4, and transferred to Group 1 an animal that had been randomized to Group 4. This transfer, which was based on the history of repeated self-inflicted catheter damage occurring in the animal originally assigned to Group 1, was undertaken to minimize additional reductions to the protocol-specified number of animals in Group 1 and occurred prior to the administration of any vehicle or test article for that iteration.

Study personnel who administered RDV or vehicle treatments and assigned clinical disease scores (which include the decision to euthanize) were experimentally blinded to the group assignment of each animal. In addition, personnel involved with in-life animal care through necropsy were blinded to telemetry data during the in-life portion of the study. The anatomic pathologist was blinded throughout the in-life phase, necropsy, and histological scoring.

Animal husbandry, including housing, food, hydration, and enrichment, was conducted as previously described [15].

### 2.4. Challenge Agent History, Propagation, and Characterization

The EBOV challenge agent used for this study, Ebola virus H. sapiens-tc/ZAI/1995/Kikwit (order *Mononegavirales*, family *Filoviridae*, genus *Orthoebolavirus*, species *zairenes*), lot number R4415, a 7U variant of EBOV (i.e., a variant with 7 consecutive template uridines at the editing site), was exactly as described previously [15].

### 2.5. Test and Control Articles

RDV (lot EW1602A1) was supplied by Gilead Sciences as a lyophilized powder containing 3.23% *w*/*w* RDV and 96.77% *w*/*w* sulfobutylether-β-cyclodextrin (SBE-β-CD). The control article, SBE-β-CD (lot EW1601A1), was supplied as a lyophilized formulation. To reconstitute the test article, 29 mL of sterile water for injection, USP, was added to a 150 mg vial of lyophilized RDV, resulting in a solution containing 5 mg/mL (nominal) RDV and 150 mg/mL (nominal) SBE-β-CD. To reconstitute the control article, 29 mL of sterile water for injection, USP, was added to a 150 mg vial of lyophilized SBE-β-CD, resulting in a solution containing 150 mg/mL (nominal) SBE-β-CD.

Dosing solutions were prepared by diluting the reconstituted vehicle or test article in 0.9% sodium chloride for injection, USP, to the appropriate concentration. To maintain experimental blinding, doses of test article and vehicle were formulated such that, regardless of group, the dosing volume was 5 mL/kg for each loading dose (first dose) and 2.5 mL/kg for each maintenance dose (all other doses). The initiation of administration of the admixed solutions occurred within 24 h of the reconstitution of vials. Dosing solutions were prepared on the basis of each animal’s body weight, determined on Day 0 prior to virus exposure. Prepared doses were stored at ambient temperature from the time syringes were prepared until the administration of doses. All doses were administered as 30 min IV infusions through the femoral CVC using a syringe pump.

Concentration verification of frozen-stored dosing solutions was conducted by Labcorp Early Development Laboratories (Madison, WI, USA) using high-performance liquid chromatography. Dose formulation analysis was performed on triplicate samples obtained for each dosing solution prepared for Day 4 PI (first dose), Day 5 PI (first maintenance dose), Day 10 PI, and the last day of dosing, Day 15 PI. Vehicle dosing solutions showed no detectable concentration of RDV; concentrations of RDV in Group 2–4 dosing solutions ranged from 88.8% to 106.1% of the target concentration.

### 2.6. Clinical Observations

Animals were evaluated daily, beginning upon movement into ABSL-4, 8 days prior to challenge and continuing through the end of the in-life phase (Table 2), for responsiveness and other signs of illness. Observations were conducted as described previously [15]. Briefly, at each awake (unanesthetized) observation, the responsiveness of each animal was scored as follows: 0 (alert, responsive, normal species-specific behavior), 1 (slightly diminished general activity, subdued, but responds normally to external stimuli), 2 (withdrawn, may have head down, upright fetal posture, hunched, reduced response to external stimuli), 3 (prostrate but able to rise if stimulated, or dramatically reduced response to external stimuli), and 4 (persistently prostrate, severely or completely unresponsive). Observations were conducted once daily while all animals scored 0. Once any animal scored ≥1, observations of all animals occurred every 3–7 h with up to 5 observations per day. Observations were completed prior to blood collection events or treatment administration.

Physical exams of animals under anesthesia occurred after awake observations on Day 0 (prior to exposure); on Days 16, 21, and 28 PI; when an animal succumbed or was euthanized; and when catheter troubleshooting procedures were required. Animals were anesthetized using an IM dose of 8–12 mg/kg ketamine. Anesthetized animals were weighed (without jackets) and evaluated for signs of illness, including rash, bleeding, discharge, swelling, lymphadenopathy, oral abnormalities, dehydration, and inoculation site changes.

Other than scheduled anesthetized exams, the use of anesthesia was minimized. No animal was anesthetized on more than 3 consecutive days or when assigned a responsiveness score ≥ 2. Animals were anesthetized for (a) blood collection when catheters were nonpatent (11 animals on one or more occasions each) or (b) the replacement of damaged tether assemblies (2 animals on one occasion each) or repair of damaged backpack and catheter lines (1 animal on two occasions).

### 2.7. Body Temperature and Activity Monitoring by Telemetry

DSI M00 telemetry devices were used to monitor body temperature and activity continuously (1 sample per second), as previously described [15]. Baseline data were collected for 5 days before challenge (Days −5 through −1). Sustained (>2 h) body temperature changes (compared to baseline values) were defined as follows: significant increase or decrease in body temperature (>3 standard deviations above or below baseline); fever (>1.5 °C above baseline); hyperpyrexia (>3.0 °C above baseline); and hypothermia (>2.0 °C below baseline). Additional parameters assessed included ∆T_Max_ (the largest ∆T value per 24 h time period), TE_Sig_ (the percentage of the 24 h daily time period during which body temperature was significantly increased), and fever-h (the sum of the significant temperature increases). Twelve-hour and six-hour activity values were considered significantly different from baseline if they were at least 3 standard deviations above or below their concomitant baseline values.

For animals found deceased, the time of death was estimated from the time at which the activity level dropped to a minimum value (signifying an absence of movement), coinciding with a continuous drop in body temperature.

### 2.8. Clinical Pathology

Blood was collected via the jugular CVC or venipuncture as described previously [15] and according to the study schedule (Table 2).

Coagulation parameters—prothrombin time, activated partial thromboplastin time (APTT), fibrinogen, thrombin time, D-dimer, and antithrombin—were assessed in plasma using a Sysmex CA-1500 (Siemens, Munich, Germany). Serum chemistry was analyzed using a Piccolo Xpress (Zoetis, Parsippany–Troy Hills, NJ, USA) for sodium, potassium, chloride, alanine aminotransferase, aspartate aminotransferase, alkaline phosphatase, total bilirubin, calcium, blood urea nitrogen (BUN), creatinine, total protein, albumin, glucose, and total carbon dioxide. Hematology analysis was conducted using an Abaxis Vetscan^®^ HM5 Hematology Analyzer (Zoetis) to assess the following parameters: neutrophils, lymphocytes, monocytes, red blood cell count (RBC), hemoglobin, hematocrit, mean corpuscular volume, mean corpuscular hemoglobin, mean corpuscular hemoglobin concentration, RBC distribution width—standard deviation, RBC distribution width—coefficient of variation, platelet count, and mean platelet volume.

### 2.9. Plasma Viral RNA Assessment

A validated quantitative reverse-transcription polymerase chain reaction (RT-PCR) was used to quantify the systemic (plasma) concentration of EBOV RNA in samples as previously described [15]. Briefly, after inactivation with TRIzol^®^ LS, samples were prepared and run on an ABI 7500 Fast Dx (Thermo Fisher Scientific, Waltham, MA, USA). Data were acquired and analyzed using ABI 7500 FAST SDS v1.4 (Thermo Fisher Scientific). Samples for which ≥ 2 replicates were below the limit of detection (LOD) of the assay (38.07 cycle threshold), reported as “<LOD”, were imputed as 3 log_10_ ge/mL. Samples for which the analyzed value was above the LOD but below the lower limit of quantitation (LLOQ) were reported qualitatively (as “>LOD, <LLOQ”) and were imputed as the LLOQ (4.903 log_10_ ge/mL). Samples for which the reported value was above the LLOQ were reported quantitatively. The upper limit of quantitation (ULOQ) was 11.903 log_10_ ge/mL; no values exceeded the ULOQ.

### 2.10. Plaque Assay for Challenge Backtiter and Viremia Assessment

Plaque assays were used to assess the challenge material and the burden of infectious virus in serum samples collected according to the study schedule (Table 2). Briefly, frozen serum was thawed at ambient temperature and diluted using a 10-fold dilution series, starting with a 1:10 dilution, in MEM containing cell culture supplements. Vero cell monolayers in six-well plates were treated with 100 µL of diluted serum in duplicate for 1 h ± 10 min, after which cells were overlaid with EBME-containing agarose. Plates were incubated under standard cell culture conditions for 8 days to allow for plaque formation. A secondary agarose overlay containing neutral red was applied to each well for 36 ± 12 h to aid in plaque visualization and enumeration. The determination of pfu per volume of serum was conducted for each sample using the plaque number obtained from the least dilute serum sample for which plaques could be counted. Plaque counts <10 and >150 per well were considered unreliable and were excluded from analysis. The LOD for this assay is 1.699 log_10_ pfu/mL (50 pfu/mL), equivalent to 1 pfu detected in one of two wells. The LLOQ for the plaque assay is 3.00 log_10_ pfu/mL (1000 pfu/mL), equivalent to 10 pfu detected in two of two wells. Values below the LOD were imputed as 1.6 log_10_ pfu/mL; values above the LOD but less than the LLOQ were imputed as 2.9 log_10_ pfu/mL.

### 2.11. Viral Genomic Sequence Analysis

Plasma samples were collected for EBOV whole-genome sequencing, inactivated with TRIzol^®^ LS, and stored frozen (−60 °C to −90 °C) prior to analysis. Viral genomic sequence analysis was performed on samples from Day 4 PI and later that were found to be positive for EBOV RNA by PCR analysis. EBOV RNA was extracted from positive samples using a PureLink RNA Mini Kit (Thermo Fisher Scientific), and viral cDNA libraries were generated according to a KAPA Hyper Prep Kit (Roche, Basel, Switzerland). The cDNA libraries were PCR amplified, and EBOV-specific sequences were extracted through a two-step hybridization protocol using EBOV 80mer oligos mapping to the reference sequence genome generated by Illumina (San Diego, CA, USA). Libraries were pooled to equimolar volumes and sequenced on the Illumina HiSeq 2500 System. Viral sequences from study animals were compared with the reference sequence of the viral inoculum (Ebola virus H.sapiens-tc/COD/1995/Kikwit challenge stock R4415; GenBank accession number KT762962) to determine whether amino acid substitutions had emerged in the L gene during RDV treatment [16]. An amino acid substitution was considered to have developed if it was detected in the virus from study animals but was not present in the reference sequence of the viral inoculum.

### 2.12. Plasma Bioanalysis and Pharmacokinetic Analysis

Whole blood was collected for plasma bioanalysis and PK analysis, and samples were stored—in K2-EDTA tubes pretreated with 80 mM dichlorvos (aqueous solution) at a ratio of 25 µL dichlorvos to 1000 µL whole blood—on wet ice or a chilled cryorack for no more than 2 h from the time of collection until initiation of centrifugation. All samples were centrifuged in a refrigerated centrifuge set for 2000× *g* for 10 min. Plasma was transferred into new polypropylene tubes and transferred to frozen storage (−60 to −90 °C) prior to virus inactivation and sample extraction.

For plasma bioanalysis, plasma was analyzed to determine concentrations of RDV and its two major systemic metabolites, GS-441524 and GS-704277. Samples were collected from all animals (survival permitting) on Days 4 and 10 PI, including pre-dose (Day 10 PI only) and approximately 0.3, 1, 2, 12, and 24 h post-dose. Systemically observed GS-704277 (intermediate alanine metabolite) is primarily formed following plasma ester hydrolysis of RDV (GS-5734), the prodrug. GS-441524, on the other hand, appears in the systemic circulation following a complex intracellular metabolic pathway that kinetically favors the formation of the active antiviral nucleoside triphosphate metabolite. Plasma samples were assayed for GS-5734, GS-441524, and GS-704277 using a bioanalytical method consisting of liquid chromatography coupled with tandem mass spectrometry.

Inside cells, RDV also undergoes hydrolysis to yield GS-704277, which then forms the nucleoside monophosphate via phosphoramidase cleavage. This monophosphate metabolite is subsequently anabolized to the kinetically favored active antiviral nucleoside analog triphosphate. The less kinetically favored metabolism of these phosphorylated metabolites results in the formation of the nucleoside metabolite, GS-441524, which is then transported back into the systemic circulation.

PK analysis was performed using Phoenix^®^ WinNonlin^®^ version 8.1 (Certara USA, Inc., Princeton, NJ, USA). Nominal doses and sampling times were used unless deviations were noted. Descriptive statistics and ratios were calculated using WinNonlin version 8.1. Concentration values below the LLOQ (2.00 ng/mL for GS-5734 and GS-441524 and 5.00 ng/mL for GS-704277) were treated as zero for descriptive statistics and PK analysis. However, if ≥50% of the group had measurable concentrations within a time point, the mean of all measured values was used for descriptive statistics. If <50% of the group had measurable concentrations within a time point, the mean value was reported as zero, and the standard deviation and percentage coefficient of variation were reported as not applicable (NA). Embedded values below the LLOQ were excluded from descriptive statistics and PK analysis. Since pre-dose samples were not collected on Day 4 PI, the pre-dose concentrations were assumed to be equal to zero. Profiles that were all below the LLOQ were excluded from PK analysis. At least three measurable concentrations were needed to report the area under the concentration–time curve (AUC), and at least two measurable concentrations after dosing were needed to report the concentration at the last quantifiable time point and the last time point at which quantifiable drug could be measured. For the calculation of descriptive statistics for PK parameters, in cases with two reportable values, a mean was reported; in cases with three or more reportable values, standard deviation and the coefficient of variation were also reported. In instances with only one reportable value (i.e., if the others were treated as missing or NA), no descriptive statistics were performed, and they were reported as NA.

### 2.13. Euthanasia and Terminal Procedures

Animals declared moribund (assigned a responsiveness score of 4) and those surviving to the scheduled end of the in-life phase of the study were euthanized via the administration of a pentobarbital-based euthanasia solution (0.3–0.4 mL/kg) via CVC or venipuncture under deep anesthesia with Telazol (6–9 mg/kg IM). Blood samples were collected as part of the terminal procedures for all animals, except those found deceased (1 animal) or euthanized on a day on which a scheduled blood collection event had already occurred. A narrative description of the clinical status of each animal at the time deemed moribund was documented prior to the administration of anesthesia. The final physical exam occurred prior to the administration of the euthanasia solution.

### 2.14. Necropsy and Histopathological Examination

Necropsies were conducted as described previously [15] using the latest version of Pristima (7.4). Tissues collected and subjected to gross and microscopic examination included lung, aorta, esophagus, trachea, heart, liver, spleen, kidney, urinary bladder, skin (with rash if present), inoculation site, adrenal gland, lymph nodes (axillary, inguinal, mesenteric, and tracheobronchial), stomach, small intestine, large intestine, pancreas, brain, eye, skeletal muscle, sciatic nerve, uterus, ovary, prostate gland, testes, and epididymis. Immunohistochemistry (IHC) and in situ hybridization (ISH) were conducted as described previously [15].

### 2.15. Statistical Analyses

A comparison of survival rates in RDV-treated groups versus vehicle control animals was conducted using a one-sided Fisher’s exact test (with survival defined as surviving 42 days after EBOV exposure). A predefined stepdown approach was used to control for multiple comparisons as follows: Survival in Group 3 (10/5 mg/kg RDV) and Group 1 (vehicle control) was compared at a one-sided significance level of 0.05. If the difference between Group 3 and Group 1 was statistically significant, survival in Group 4 (20/10 mg/kg RDV) and Group 1 (vehicle) was to be compared at a one-sided significance level of 0.05. If this difference was statistically significant, survival in Group 2 (5/2.5 mg/kg RDV) and Group 1 (vehicle) was to be compared at a one-sided significance level of 0.05. Survival analysis was conducted using the Kaplan–Meier method. Survival curves were also compared between the vehicle group and each RDV treatment group using a log-rank test.

The EBOV RNA (log_10_ ge/mL) values by day were summarized using descriptive statistics for each treatment group. Pairwise comparisons of EBOV RNA (log_10_ ge/mL) values for each RDV treatment group were made against the vehicle control group using analyses of variance or nonparametric methods. For clinical laboratory parameters, pairwise comparisons of each RDV treatment group were made against vehicle using the Wilcoxon rank-sum test for the value at each study day and the change from Day 0 (day of challenge) to each Day PI. Comparisons of the changes from Day 0 within a treatment group were made using the Wilcoxon signed-rank test for each Day PI.

## 3. Results

### 3.1. Survival

**Figure 1:**
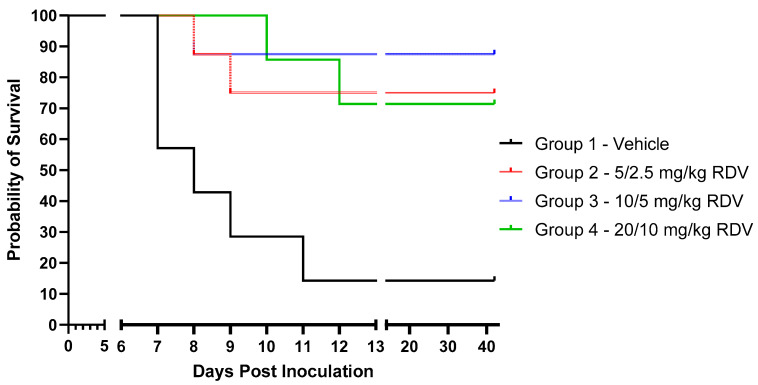
Kaplan–Meier plot of animal survival in each treatment group.

Of the seven vehicle control animals (Group 1), only one (14.3%) survived, with mortality events occurring on Days 7–11 PI (Figure 1; Table 3). The surviving vehicle control animal exhibited plasma viral RNA and numerous disease signs consistent with EVD before recovering. Of the eight animals in Group 2 (5/2.5 mg/kg RDV), six (75%) survived to the end of the in-life phase, with mortality events occurring on Days 8 and 9 PI in animals that succumbed. Seven of eight animals (87.5%) in Group 3 (10/5 mg/kg RDV) survived; the single nonsurviving animal in Group 3 succumbed on Day 8 PI. In Group 4 (20/10 mg/kg RDV), five of seven animals (71.4%) survived, with mortality events in this group occurring on Days 10 and 12 PI.

All mortality events occurred via euthanasia, except for one animal in Group 1 (vehicle control), which was found deceased upon first observation on Day 11 PI. Of the euthanized animals, all but one (Group 4) were euthanized upon meeting protocol-specified euthanasia criteria (assignment of a responsiveness score of 4).

An unanticipated event occurred involving one animal in Group 4 (20/10 mg/kg RDV), which prompted the animal’s euthanasia. Through samples obtained on Day 9 PI, this animal was observed to have highly elevated levels of serum BUN and creatinine: 145 mg/dL and 13.1 mg/dL, respectively. Samples obtained on Day 11 PI showed continued increases, with the concentration of both analytes exceeding the instrumentation limits: >180 mg/dL BUN and >20 mg/dL creatinine. Consultations were held with test facility veterinarians and separately with the sponsor. The animal was euthanized for humane reasons based on BUN and creatinine findings consistent with acute kidney failure. At the time of euthanasia, the animal was assigned a responsiveness score of 3. While other clinical pathology values were indicative of EVD, the dramatic elevations of BUN and creatinine are not considered typical of EVD. Based on the design of this study (all animals were challenged with EBOV), it is not possible to attribute definitively these atypical findings to a particular cause based on available information. Effects on the kidney may be related, at least in part, to high-dose RDV treatment. This animal has been included in the survival analyses and is considered a treatment failure. All other mortality events are attributed to EBOV infection.

Based on the predefined stepdown procedure, animals in Group 3 (10/5 mg/kg RDV) showed a significantly improved (*p* = 0.009) survival rate compared to the vehicle-treated control animals in Group 1. While improved survival trends were observed in RDV-treated Groups 4 and 2 compared to vehicle, these differences in survival rates lacked statistical significance based on the stepdown procedure (*p* = 0.051 and *p* = 0.032, respectively; Table 3).

### 3.2. Responsiveness

**Figure 2:**
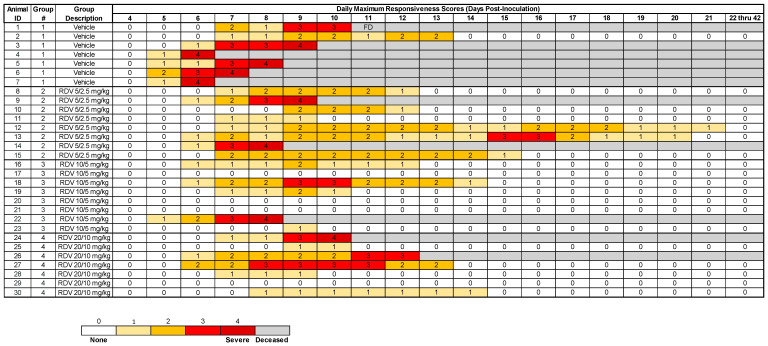
Schematic of daily maximum responsiveness scores by individual animal. All animals were assessed daily and assigned a score of physical signs and responses based on the following criteria: 0 = alert, responsive, normal species-specific behavior; 1 = slightly diminished general activity, subdued, but responds normally to external stimuli; 2 = withdrawn, may have head down, upright fetal posture, hunched, reduced response to external stimuli; 3 = prostrate but able to rise if stimulated or dramatically reduced response to external stimuli; 4 = persistently prostrate, severely or completely unresponsive. The scores shown are the highest for each day. Animals were considered moribund and were euthanized upon assignment of a responsiveness score of 4. One animal in Group 4 was euthanized due to serum chemistry findings, consistent with kidney failure despite a responsiveness score of 3. One animal in Group 1 was found deceased (FD).

The onset of clinically observed reduced behavioral activity and change in posture (i.e., assignment of a responsiveness score ≥ 1) in one or more animals first occurred on Day 5 PI (Figure 2; Table S1).

Group 1 (vehicle) animals were first assigned responsiveness scores ≥ 1 on Days 5–7 PI, with seven of seven animals developing signs of illness. Five of seven animals were assigned the maximum responsiveness score of four and were euthanized. One Group 1 animal was found deceased after reaching a maximum score of 3, and one animal survived. For the single surviving Group 1 animal, the maximum responsiveness score was 2; the responsiveness scores for this animal returned to 0 from Day 13 PI through the end of the study.

Group 2 (5/2.5 mg/kg RDV) animals were first assigned responsiveness scores ≥ 1 on Days 6–9 PI, with eight of eight animals developing disease signs. Two of eight animals were euthanized after reaching a score of 4. The maximum responsiveness scores for the six surviving animals ranged from 1 to 3; the scores returned to 0 by Day 21 PI.

In Group 3 (10/5 mg/kg RDV), three of eight animals remained free of disease signs (were assigned a responsiveness score of 0 at all observation events). Among the other five Group 3 animals, disease signs were first observed on Days 5–9 PI. One of eight animals reached a score of 4 and was euthanized, and seven animals survived. The maximum responsiveness scores for the seven surviving animals ranged from 0 to 3, and disease signs resolved in all Group 3 animals by Day 14 PI.

In Group 4 (20/10 mg/kg RDV), one of seven animals remained free of disease signs. Six animals developed disease signs on Days 6–9 PI. One animal reached a score of 4 and was euthanized. One animal was euthanized by veterinary decision on Day 12 PI after exhibiting BUN and creatinine levels consistent with acute kidney failure, and five animals survived. The maximum responsiveness scores for the five surviving animals ranged from 0 to 3, and the scores resolved by Day 14 PI.

### 3.3. Plasma Viral RNA

**Figure 3:**
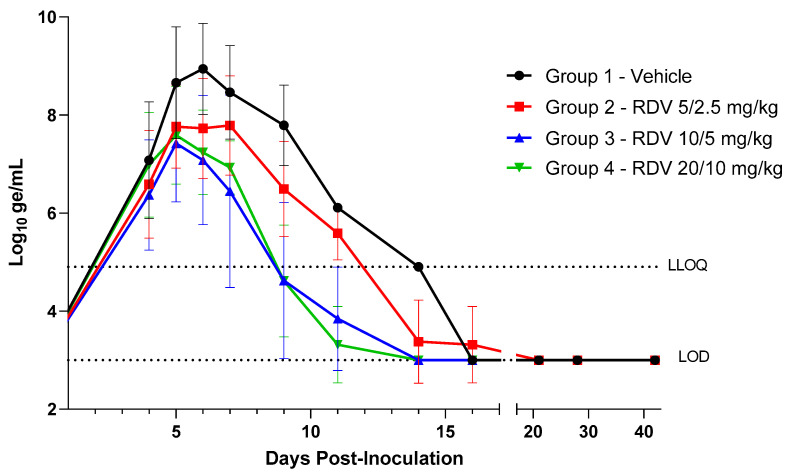
Group mean plasma viral RNA over time. Vertical bars show the standard deviation. Dotted lines show the limit of detection (LOD = 3 log_10_ ge/mL) and the lower limit of quantitation (LLOQ = 4.903 log_10_ ge/mL). For display and analyses, EBOV RNA values below the LOD were imputed as 3 log_10_ ge/mL; values above the LOD but below the LLOQ (“>LOD, <LLOQ”) were imputed as 4.903 log_10_ ge/mL. The X-axis has been truncated to highlight responses during the acute phase of disease. Data for days on which only terminal samples were obtained from animals that succumbed are not included.

Plasma viral RNA was detected via RT-PCR in all animals at the first sampling event on Day 4 PI, taken immediately prior to the first vehicle or RDV treatment (Figure 3; Table S1). Individual values at this time ranged from 5.01 to 8.67 log_10_ ge/mL, and group means ranged from 6.37 (Group 3) to 7.08 log_10_ ge/mL (Group 1).

In the vehicle control group, mean viral RNA concentration increased from Day 4 PI (7.08 log_10_ ge/mL) to Day 5 PI (8.66 log_10_ ge/mL), reaching a maximum on Day 6 PI (9.01 log_10_ ge/mL). In samples obtained on Day 5 PI, 1 day after vehicle and RDV treatments were initiated, the mean viral RNA was lower in all RDV-treated groups compared to the vehicle control, although differences were significant only in the 10/5 mg/kg RDV animals (Group 3; *p* = 0.031). On Day 6 PI, 2 days after initiation of vehicle and RDV treatments, mean viral RNA concentrations were significantly lower in all RDV-treated groups compared to the vehicle control, with mean values ranging from 7.08 log_10_ ge/mL (Group 3; 10/5 mg/kg RDV) to 9.01 log_10_ ge/mL (Group 1; vehicle). On Day 7 PI, mean viral RNA concentration was 8.47 log_10_ ge/mL in the vehicle control, and lower mean values were observed in all RDV-treated groups compared to vehicle, although the difference was significant only for Group 3 (10/5 mg/kg RDV; *p* = 0.023). Meaningful statistical comparisons after Day 7 PI were precluded by mortality events occurring primarily in the vehicle control group. All viral RNA samples were below the LLOQ after Day 11 PI.

### 3.4. Serum Infectious Virus Load

No infectious virus was detected by plaque assay in any serum samples prior to challenge (Day 0) or for any of the 19 surviving animals on Days 21, 28, and 42 PI, consistent with recovery (Table 4).

Infectious virus ranged from 5.13 log_10_ pfu/mL to 6.95 log_10_ pfu/mL in terminal samples from five of the vehicle control (Group 1) animals that succumbed to disease (a terminal sample was not collected for the single animal found deceased). For the vehicle control animal that survived the challenge, infectious virus within serum was above the LOD on Days 7 and 9 PI but never rose above the LLOQ on any of the collection days.

In the 5/2.5 mg/kg RDV animals (Group 2), serum infectious virus was greater than the LLOQ in only three of the samples, including two collected on Day 7 PI and one terminal sample on Day 8 PI. However, three animals had detectable virus (>LOD, <LLOQ) in the serum on Day 7 PI, and three animals had detectable virus on Day 9 PI.

For Group 3 (10/5 mg/kg RDV), the only serum sample with serum infectious virus values above the LLOQ was for the single animal from this group that succumbed to the infection (4.41 log_10_ pfu/mL on Day 7 PI). Samples from four animals had serum plaque assay values that were above the LOD but below the LLOQ on either Day 7 or Day 9 PI. The remaining three animals did not have plaques detected in serum from any time point.

Five of seven animals in Group 4 (20/10 mg/kg RDV) had serum infectious virus values above the LOD but below the LLOQ on Day 7 PI. Only one of seven animals had detectable serum infectious virus (>LOD, <LLOQ) on Day 9. The two animals from this group that succumbed had detectable serum infectious virus in terminal samples.

### 3.5. Viral Genomic Sequence Analysis

Sequencing results for the L gene were available for 100% (twenty-three of twenty-three) of the RDV-treated and 100% (seven of seven) of the vehicle control animals for at least one time point. Amino acid changes were observed in five of twenty-three and one of seven animals receiving RDV and vehicle control, respectively. None of the amino acid changes were observed in more than one RDV-treated animal. The observed amino acid changes were located outside of the polymerase active site and are not expected to be associated with RDV resistance.

### 3.6. Plasma Bioanalysis and Pharmacokinetic Analysis

**Figure 4:**
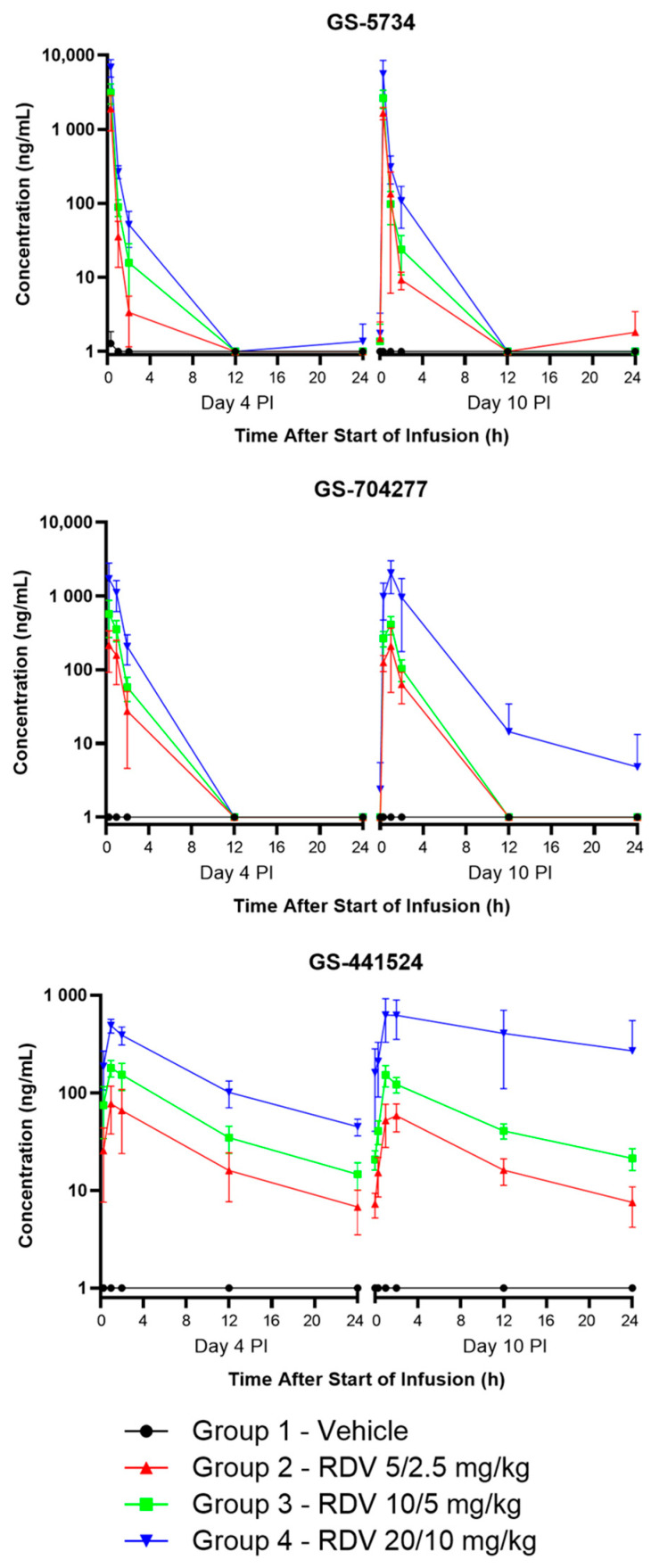
Group mean GS-5734, GS-704277, and GS-441524 concentrations in rhesus monkey plasma samples, as measured using liquid chromatography coupled with tandem mass spectrometry. Vertical bars show the standard deviation.

Plasma was analyzed via liquid chromatography coupled with tandem mass spectrometry to determine concentrations of RDV and its two major systemic metabolites, GS-441524 and GS-704277 (Figure 4 and Table 5).

Plasma exposure to RDV, GS-704277, and GS-441524 increased with dose level on Day 4 PI (loading doses) and Day 10 PI (maintenance doses). The mean plasma AUC metabolite to parent ratios indicated that RDV was efficiently converted to GS-704277 and ultimately yielded GS-441524 following IV infusion (Table 5).

Several notable anomalies were observed. GS-5734, GS-441524, and GS-704277 were below the LLOQ for one animal (a nonsurvivor in Group 2) at all time points assayed (Day 4 PI time points at 0.3, 1, 2, 12, and 24 h). The patency of the femoral catheter used for drug delivery for this animal could not be verified by pulling out blood. Thus, the catheter was “push-only”, and this was noted on all treatment days. Numerous other animals in the study, both survivors and nonsurvivors, across all groups also had “push-only” femoral catheters (21 of 30 animals on Day 4 PI), but a measurable drug was obtained in the other Group 2, Group 3, and Group 4 animals with “push-only” catheters, and there is no evidence that delivery was diminished in these animals. Upon necropsy, it was noted that this animal had a small clot at the tip of the catheter. Thus, it is possible that while the drug was being pushed into the catheter, it was released via a leak into the jacket area, which would not have been easily observed by the cage-side technical team. Since the bioanalysis and PK analysis were performed only on Day 4 PI for this animal, which did not survive to Day 10 PI, it is unknown whether RDV delivery was also impeded on the subsequent treatment days.

### 3.7. Body Temperature and Activity

Body temperature and activity were assessed via telemetry. In these temporally fine-grained assessments, body temperature and activity events measured by telemetry are typically described using “days” that are defined as 24 h periods measured from the actual time of challenge rather than by calendar day. Where the calendar day is meant (with exposure on Day 0), this is denoted as “Day x PI”, as it is elsewhere in this report.

#### 3.7.1. Body Temperature

**Figure 5:**
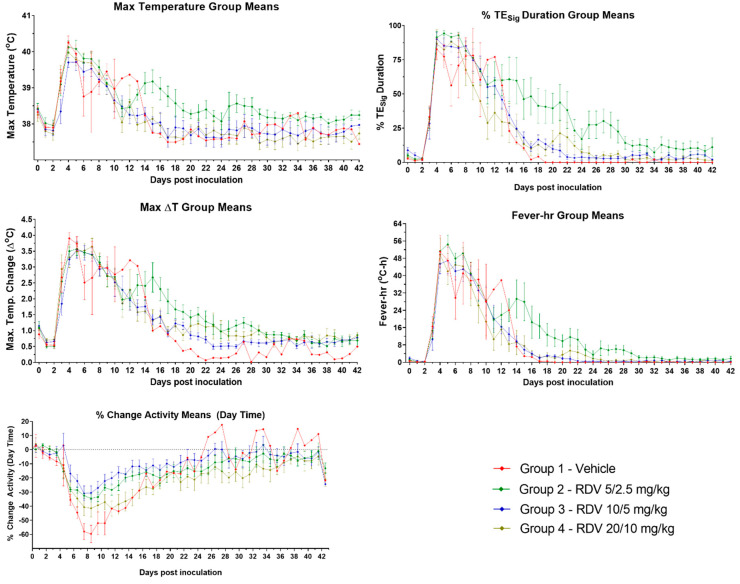
Body temperature and activity measured by telemetry. Days post-inoculation are calendar days. Vertical bars in all figures represent the standard error of the mean. Max ∆T, or maximum daily temperature elevation, is the largest change in temperature value for the 24 h daily time period. % TE_Sig_ duration is the percentage of the 24 h daily time period during which body temperatures were significantly elevated.

Notable body temperature findings are summarized in Table S1 and Figure 5. Compared with animals in the RDV treatment groups, vehicle control (Group 1) animals showed fewer significant body temperature elevations (>3 standard deviations above concomitant baseline values for >2 h) and fewer episodes of fever (>1.5 °C above concomitant baseline values for >2 h). Exceptions to this pattern were one animal that was removed from the study on Day 4 PI due to catheter failure and the Group 1 survivor. The onset of significant temperature elevation among Group 1 animals was between 3.00 and 3.65 days after challenge. The surviving animal had a prolonged period (from 3.94 to 13.44 days after inoculation) of significant temperature elevation and episodes of fever, disrupted by a 3.5 h isolated episode of decreased temperature (>3 standard deviations below concomitant baseline values for >2 h) at 8.75 days after challenge prior to returning to a normal body temperature pattern. Five animals also showed episodes of hyperpyrexia (>3.0 °C above concomitant baseline values for >2 h).

Animals in Group 2 (5/2.5 mg/kg RDV) exhibited a high frequency of significant body temperature elevations and episodes of fever throughout the duration of the study, with the exception of two nonsurviving animals that met euthanasia criteria on Day 9 PI and Day 8 PI. The onset of significant temperature elevation was between 3.08 and 3.71 days after challenge for all animals in this group. Surviving animals continued to exhibit episodes of significant temperature elevation after Day 13 PI at different rates. Periods of hyperpyrexia were observed in six animals.

Animals in Group 3 (10/5 mg/kg RDV) exhibited less frequent significant temperature elevation and fever events compared to the 5/2.5 mg/kg RDV group. By Day 15 PI, the body temperature patterns and temperature changes returned to a normal range for all animals compared to their corresponding baseline values, with very few episodes of significant temperature elevation and no episodes of fever for the remainder of the study in the surviving animals. The onset of significant temperature elevation was between 2.58 and 3.75 days following challenge for all animals, with the exception of one animal that showed an unexplained 3 h period of significant temperature elevation at 0.02 days after inoculation. Periods of hyperpyrexia were exhibited by five animals.

Animals in Group 4 (20/10 mg/kg RDV) showed a pattern of significant temperature elevations and fever events similar to that of the 10/5 mg/kg RDV group, with the exception of the two nonsurviving animals. The onset of significant temperature elevation was between 2.75 and 5.10 days after challenge for all Group 4 animals. Periods of hyperpyrexia were observed in four animals.

#### 3.7.2. Activity

The mean percentage change in activity by group is summarized in Figure 5.

The 12 h activity averages, starting at 0600 on Day 0 (day of EBOV challenge), were analyzed by comparison to baseline values. In general, animals in all groups showed decreased activity starting at approximately 3–5 days after challenge. With the exception of two animals (one each in Groups 3 and 4), this diminished activity was apparent mostly during the light periods (0600–1800) during the clinical phase of infection. Compared to light periods, dark period (1800–0600) activity reductions were not as pronounced, and dark period activity often increased during the early period of infection. Significant increases in dark period activity (≥3 standard deviations above baseline) suggest a more pronounced disruption of the sleep patterns in those animals. Surviving animals showed a general pattern of activity values returning to a level closer to baseline as early as Day 10 PI.

### 3.8. Clinical Pathology

**Figure 6:**
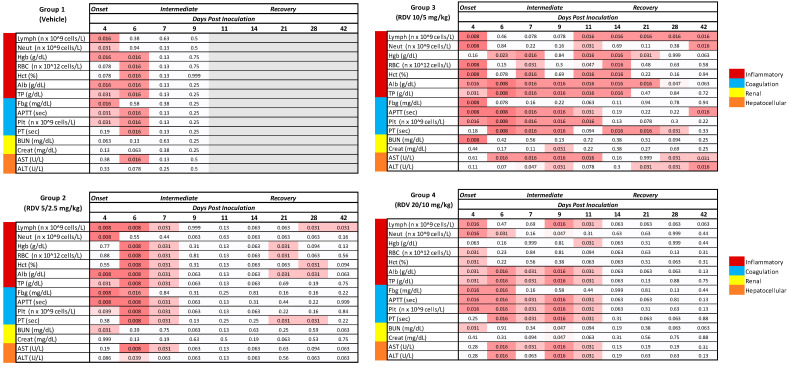
Timing of statistically significant clinicopathologic changes. The *p*-values shown represent the comparison to baseline within each group. Alb, albumin; Creat, creatinine; Fbg, fibrinogen; Hct, hematocrit; Hgb, hemoglobin; Lymph, lymphocytes; Neut, neutrophils; Plt, platelets; RBC, red blood cells; TP, total protein. White cells denote a lack of significance; pink shading is a heatmap, with deeper pink denoting greater significance. *p*-values of 0.999 are >0.999.

**Figure 7:**
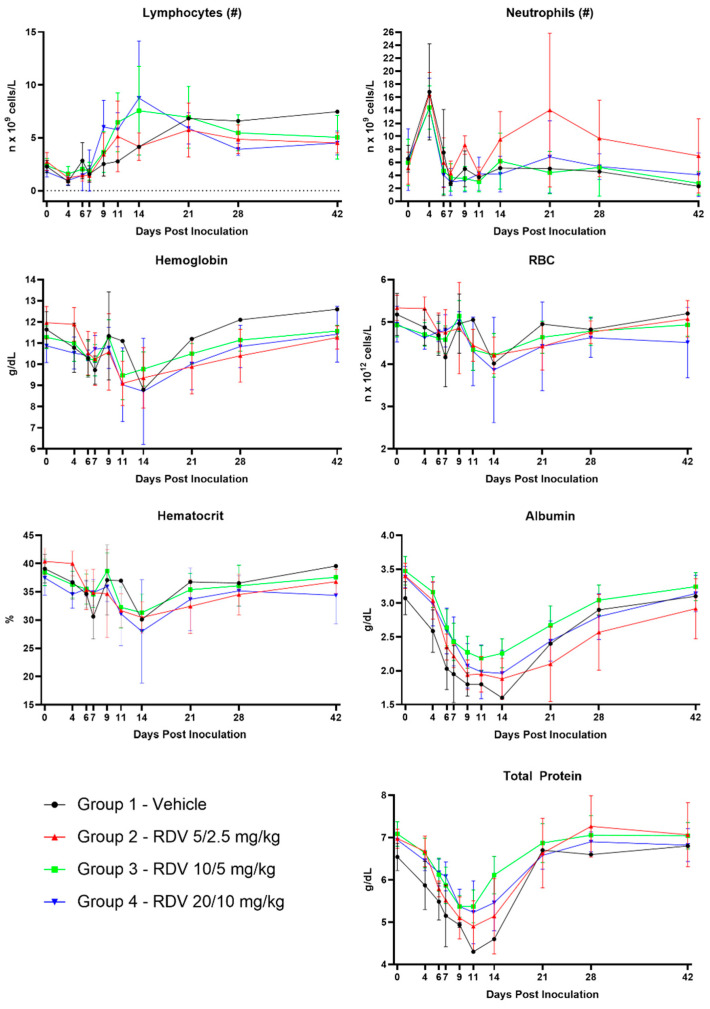
Hematological and inflammatory parameters. Figures show group means by day; vertical bars represent standard deviation. RBC, red blood cells; #, absolute numbers.

**Figure 8:**
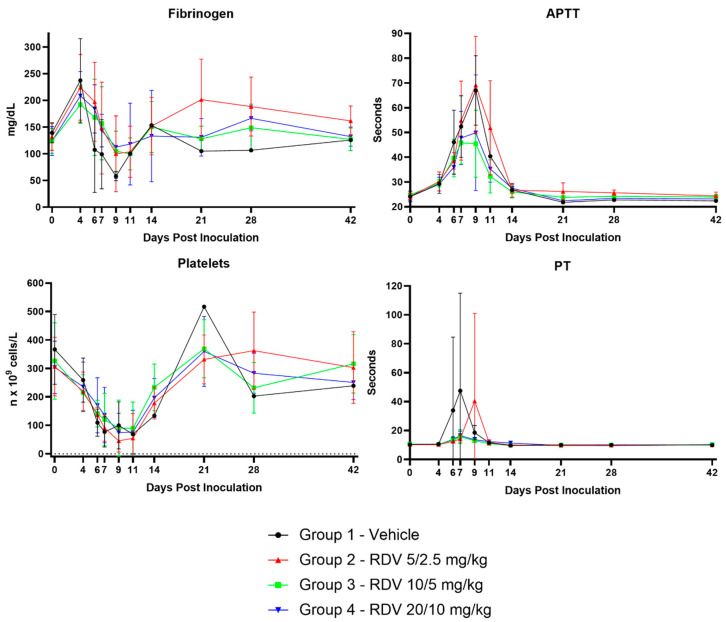
Coagulation parameters. Figures show group means by day; vertical bars represent standard deviation. APTT, activated partial thromboplastin time; PT, prothrombin time.

**Figure 9:**
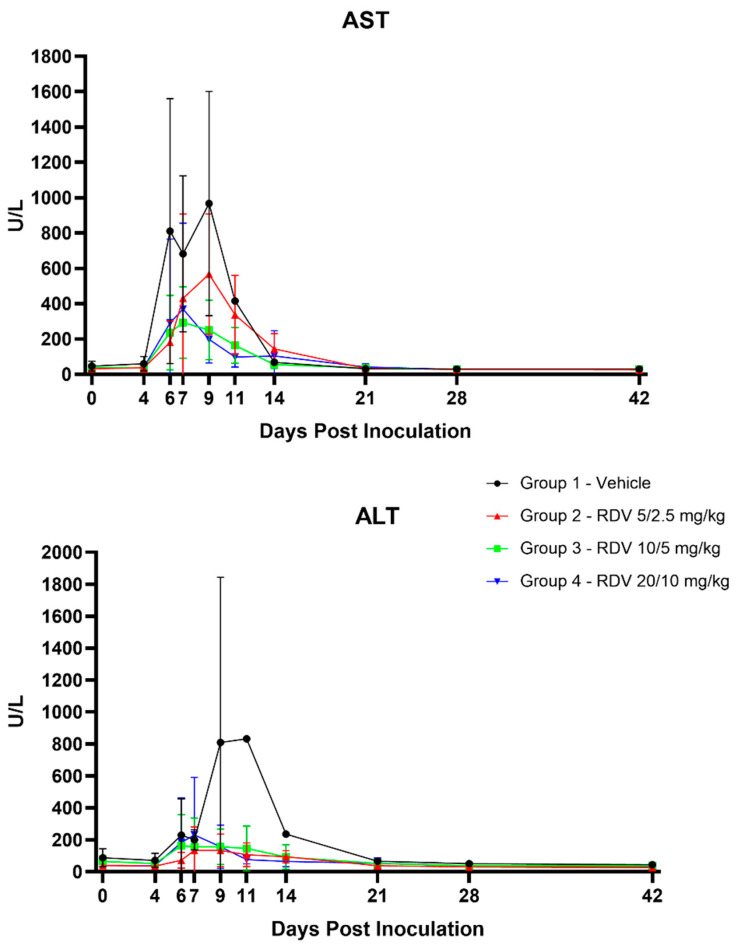
Hepatocellular parameters. Figures show group means by day; vertical bars represent standard deviation. ALT, alanine aminotransferase; AST, aspartate aminotransferase.

**Figure 10:**
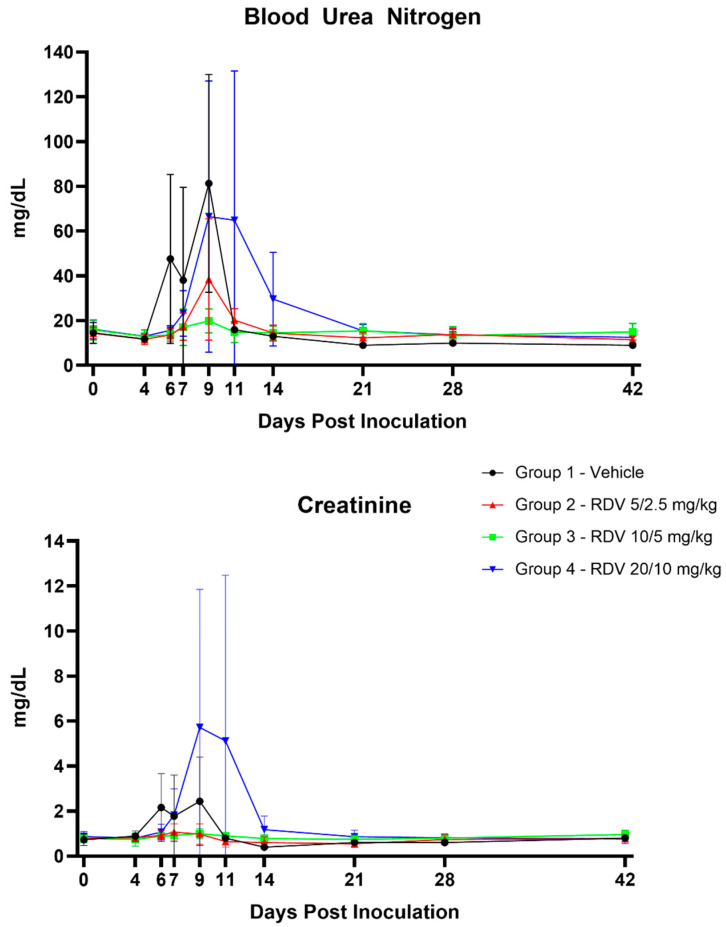
Renal parameters. Figures show group means by day; vertical bars represent standard deviation.

Figure 6 summarizes statistically significant changes in clinical pathological parameters. The key disease signs supported by the clinical pathology parameters include evidence of systemic inflammation, blood loss, changes in coagulation parameters suggestive of disseminated intravascular coagulopathy (DIC), and hepatocellular injury in all groups (Figure 7, Figure 8, Figure 9 and Figure 10). Data supporting alterations in glomerular filtration rates were generally limited to Group 1 (vehicle) and Group 4 (20/10 mg/kg RDV). Incipient EVD is suspected in all groups by Day 4 PI. Group 1 (vehicle) progressed to show evidence of fulminant disease, such as worsening clinicopathological indices and in-life observations of epistaxis, cutaneous hemorrhages, and increased daily maximum responsiveness score. Additionally, microscopic observations of hemorrhage and thrombi in multiple organs suggestive of DIC, hepatocellular necrosis, and renal tubular necrosis were observed in many animals that succumbed to disease.

RDV treatment, regardless of dose, often resulted in amelioration of many of the clinical pathology changes. Based on survival and clinical pathology endpoints, Group 3 (10/5 mg/kg RDV) appeared to be the most efficacious treatment regimen. Increases in BUN and creatinine concentrations were mitigated, and evidence of hepatocellular damage and coagulopathy was ameliorated. Lymphocyte counts also recovered more robustly than those noted in Group 1 (vehicle) or Group 2 (5/2.5 mg/kg RDV). Although speculative, this may suggest an earlier improvement in lymphocyte-associated immune function.

Group 2 treatment (5/2.5 mg/kg RDV) resulted in amelioration in a subset of clinical pathological parameters, although a few of the surviving animals in this group experienced a second occurrence of systemic inflammation based on transient increases in neutrophil numbers and fibrinogen concentration. The worsening in-life correlates in a subset of animals suggest recrudescence or reemergence of EVD. Consistent microscopic correlates were not present.

Group 4 treatment (20/10 mg/kg RDV) also resulted in amelioration in a subset of clinical pathological parameters. However, this dosing regimen may have impacted renal function in monkeys. One animal in Group 4 was diagnosed with acute renal failure during RDV treatment, with microscopic correlates of renal tubular necrosis and cellular casts noted. Additionally, a subset of animals in this group had creatinine concentrations that remained elevated longer than those of animals in other groups. Consistent microscopic correlates were not present in these animals, but azotemia had resolved in surviving animals by the end of the study, suggesting a transient impact on renal function.

### 3.9. Anatomic Pathology

Gross and microscopic findings are summarized in Table 6. Nine of the eleven nonsurviving animals (six animals in Group 1, two animals in Group 2, and one in Group 3) had gross and microscopic findings consistent with EVD, including positive signals for EBOV antigen (IHC) and EBOV RNA (ISH). The two nonsurvivors in Group 4 had fewer and/or less severe EBOV-typical macroscopic and microscopic findings with reduced or absent IHC and ISH signals. Both Group 4 nonsurvivors exhibited significant renal damage that was not consistent with EVD and likely contributed to their deaths. Surviving animals displayed generally less severe and resolving gross and microscopic findings and no IHC or ISH signal.

#### 3.9.1. Gross Findings

Of the eleven nonsurvivors; ten animals (91%) had one or more changes in the liver (pale, friable, and/or enlarged); nine animals (82%) had a skin rash; nine animals (73%) had a firm and/or enlarged spleen; eight animals (73%) had either a focal or multifocal area of redness on the gastrointestinal mucosa (hemorrhage); and four of the six males (67%) had red testes (hemorrhage). Less common findings included pale kidneys (four animals, 36%); lung lobes that failed to collapse (five animals, 45%); and red areas on the urinary bladder mucosa (two animals, 18%; Table 6).

On gross examination, no differences were observed between nonsurvivors in the RDV-treated groups and those in the vehicle group. The only noticeable outlier was one animal in Group 3 for which no gross changes were noted in the liver or spleen.

Among the nineteen survivors, seventeen animals (89%) had one or more enlarged lymph nodes, thirteen animals (68%) exhibited splenic enlargement, and four animals (21%) had some occurrence of pulmonary adhesions involving the diaphragm, pericardium, or thoracic wall.

#### 3.9.2. IHC and ISH

Among nonsurvivors in Groups 1, 2, and 3, positive IHC and ISH staining (for EBOV antigen and RNA, respectively) was identified in all examined tissues, with few exceptions. The pancreas was IHC negative for one animal in Group 2, the prostate was ISH negative for one animal in Group 1 (vehicle), and the uterus was ISH negative for one animal in Group 1. Group 4 (20/10 mg/kg RDV) nonsurvivors had reduced signal in most tissues and were negative in a few others. Both Group 4 nonsurvivors were positive for both IHC and ISH in the lung, liver, inoculation site, and pancreas. One animal was ISH negative for all remaining tissues but had additional positive IHC signal in the spleen, kidney, inguinal lymph node, and corpus striatum. One animal was also positive in the spleen, inguinal lymph node, corpus striatum, thalamus, and eye for both IHC and ISH and additionally was IHC positive in the kidney, jejunum, and ileum.

Among the survivors, six animals demonstrated positive IHC and/or ISH in a few tissues. Positive IHC and ISH were seen together only in immune-privileged sites in three animals. One animal in Group 2 (5/2.5 mg/kg RDV) and another in Group 3 (10/5 mg/kg RDV) had signal in the eye, and one animal in Group 3 was positive in the right testis and epididymis. Three animals demonstrated positive IHC signal only: one animal in Group 3 (lung and heart), one animal in Group 4 (kidney), and one animal in Group 2 (corpus striatum).

#### 3.9.3. Findings in Specific Tissues

This section provides a summary of the most notable changes in specific tissues.

##### Liver

Among the 11 nonsurvivors, 10 animals (91%) exhibited mild to moderate degeneration and necrosis with inflammation in the liver (Figure S1A,B). Minimal to mild inflammation was observed in the liver in 17 of 19 survivors (89%). One surviving animal (5%) exhibited moderate degeneration of the liver, and six survivors (32%) had minimal to mild hepatocellular necrosis.

##### Spleen

All 11 nonsurvivors (100%) exhibited moderate to severe lymphoid depletion in the spleen (Figure S1C,D). Fibrin deposition in the spleen was noted in 10 nonsurvivors (91%) with a severity of mild to severe. No surviving animals had lymphoid depletion or fibrin deposition in the spleen. However, lymphoid hyperplasia in the white pulp of the spleen was found in 15 of 19 survivors (79%) with minimal to moderate severity.

##### Kidney

Tubular epithelial degeneration and necrosis of the kidney were observed in seven of eleven nonsurviving animals (64%; Figure S1E,F). Five nonsurvivors (45%) exhibited intratubular mineral deposition in the kidney. Among survivors, only one animal of nineteen (5%) exhibited mild tubular degeneration and necrosis in the kidney. Seven survivors (37%) had intratubular mineral deposition in the kidney.

##### Lymph Nodes

Among nonsurviving animals, 10 of 11 (91%) had minimal to marked lymphoid depletion and lymphocytolysis in one or more of the collected lymph nodes (Figure S1G,H). No surviving animals exhibited lymphoid depletion, but 18 of 19 (95%) had minimal to moderate lymphoid hyperplasia.

##### Duodenum

Minimal to severe hemorrhage and necrosis were observed in the duodenum of six of eleven nonsurviving animals (55%; Figure S1I,J). No survivors exhibited necrosis or hemorrhage in the duodenum.

##### Other Microscopic Findings

Additional findings in nonsurvivors included inflammation in the lungs (eleven animals, 100%); inflammation and skeletal muscle degeneration at the site of challenge (six animals, 55%); and hemorrhage of the testes (five of six male animals, 83%). Fibrin thrombi were noted in various organs of numerous nonsurviving animals. Sites of fibrin thrombi formation or fibrin deposition included the liver, red pulp of the spleen, lung, kidney, gastrointestinal tract, and choroid plexus. Sites of hemorrhage included the lung, heart, white pulp of the spleen, kidney, inoculation (challenge) site, adrenal gland, various lymph nodes, gastrointestinal tract, testes, uterus, and brain.

Among surviving animals, inflammatory infiltrates were observed in multiple organs, including the lung, liver, kidney, stomach, urinary bladder, brain, meninges, prostate, eye, and heart. Fibrin thrombi were seen in the lungs of seven survivors. Hemorrhage was identified in three animals in the skeletal muscle at the inoculation site, skin, and adrenal gland.

The remaining microscopic findings were considered incidental or background lesions.

## 4. Discussion

The present study tested the efficacy of three RDV dosing regimens, administered by IV infusion beginning on Day 4 PI, in animals inoculated with EBOV by the IM route. Animals were administered vehicle control or RDV loading/maintenance doses of 5/2.5, 10/5, or 20/10 mg/kg once daily for 12 days.

Of the seven vehicle control animals (Group 1), one animal (14.3%) survived EBOV infection. In the RDV treatment groups, six of eight (75%), seven of eight (87.5%), and five of seven (71.4%) animals survived in Group 2 (5/2.5 mg/kg), Group 3 (10/5 mg/kg), and Group 4 (20/10 mg/kg), respectively. Using Fisher’s exact test according to a predefined multiple-comparison stepdown approach, animals receiving 10/5 mg/kg RDV (Group 3) showed a statistically significant (*p* = 0.009) improvement in survival rate compared to the vehicle-treated control animals in Group 1. While improved survival trends were observed in the 20/10 mg/kg RDV and 5/2.5 mg/kg RDV groups compared to vehicle, these differences in survival rate lacked statistical significance (*p* = 0.051 and *p* = 0.032, respectively) based on the stepdown procedure (Table 3).

Of the eleven animals that succumbed, the cause of death for ten appeared to be from typical EVD manifestations, with nine euthanized upon reaching the predetermined EBOV euthanasia criteria and one found deceased in its cage. Two animals that succumbed during the study deserve special consideration. One animal in Group 4 was considered a treatment failure since it was euthanized due to highly elevated serum BUN and creatinine. One animal in Group 2, which succumbed to fulminant EVD, likely did not receive the appropriate amount of RDV (based on PK sample analysis) and was thus considered a technical failure. Neither of these animals was excluded from any analyses, including survival analysis.

In addition to having the highest survival rate among the treatment groups, the 10/5 mg/kg treatment group also had the most animals remaining free of disease signs (three of eight). The 10/5 mg/kg RDV regimen resulted in a significantly lower systemic viral load (measured by RT-PCR) compared to vehicle control by Day 5 PI (after a single RDV treatment); by Day 6 PI, mean viral load in all three RDV-treated groups was significantly lower than that of vehicle control. Collectively, these data support the 10/5 mg/kg RDV regimen as the optimal regimen to confer protection in the IM EBOV rhesus model of EVD.

In surviving animals, gross pathology observations were consistent with resolving disease, with a majority of animals exhibiting gross changes, including enlarged lymph nodes, and microscopic changes, such as lymphoid hyperplasia of the white pulp of the spleen and/or lymph nodes (Table 6 [right panel]). Clinical pathology parameters measuring coagulopathy and hepatocellular damage were ameliorated in RDV-treated animals that survived infection as well as the Group 1 (vehicle) survivor. Finally, whereas tissues from most nonsurviving animals were IHC and ISH positive, very few IHC- or ISH-positive tissues were found in survivors. Three survivors had positive IHC (but not ISH) in tissues such as the kidney, lung, heart, and/or corpus striatum. However, in three surviving animals, the only IHC- or ISH-positive tissues were in immune-privileged sites.

One important consideration in designing this study was the RDV treatment initiation time. The intention was to begin treatment at a time when all animals were likely to show signs of incipient EVD. Day 4 PI was chosen as the start of treatment based on findings from the previously conducted EBOV natural history study [15]. In the present study, several parameters indicated that EVD was underway by Day 4 PI, prior to treatment. All 30 animals in the study had plasma samples with quantifiable levels of EBOV RNA by RT-PCR (Table S1). Additionally, prior to treatment initiation, 29 of 30 animals had significantly elevated temperature, and 26 of 30 had onset of fever (Table S1). In all groups, numerous clinical pathology parameters were significantly changed from baseline: lymphocytes, neutrophils, albumin, total protein, fibrinogen, APTT, and platelets (Figure 6).

Lessons learned through recent EBOV and SARS-CoV-2 outbreaks point to key niches for how RDV could be used to further advance the standard of care. Through the Pamoja Tulinde Maisha (PALM [“Together Saves Lives” in Kiswahili]) trial conducted in the Democratic Republic of the Congo, mAb114 and REGN-EB3 were identified as superior therapeutics with mortality rates of 34% and 35%, respectively, compared to 53% for RDV-treated patients [12]. However, since RDV and monoclonal antibodies have different mechanisms of action and distinct therapeutic penetration into immune-privileged sites, a combination therapy that includes RDV and a leading monoclonal antibody therapy has the potential to further increase survival, reduce disease sequalae, extend the therapeutic window, decrease viral persistence in immune-privileged tissue reservoirs, reduce the likelihood of sexual transmission and the ignition of new outbreaks, and prevent the development of resistance [17]. Indeed, combining a 10/5 mg/kg dose of RDV with filovirus monoclonal antibodies resulted in improved survival with an extension of the therapeutic window using nonhuman primate models of SUDV and MARV diseases [9,13]. The results of these combination studies support clinical testing of the combination of direct antiviral agents and virus-neutralizing monoclonal antibodies for any filovirus infection, as proposed in the World Health Organization–sponsored Solidarity Partners trial protocol [18]. RDV treatment of EBOV survivors also has the potential to reduce or eliminate viral reservoirs via small-molecule penetrations to immune-privileged sites such as the testis, eye, and central nervous system [14,19,20]. Veklury (the trade name for RDV) is approved for COVID-19 treatment in patients, including those with severe renal impairment, and the 10/5 mg/kg dosing regimen reported here showed GS-441524 plasma exposure similar to that of the approved 200/100 mg regimen in humans, which has an established safety profile [21]. Finally, RDV has broad-spectrum (pan-filovirus) antiviral activity, a stable shelf life, and availability, making RDV an important tool for future filovirus outbreaks.

## Figures and Tables

**Table 1:** Experimental design.

Group No.	Group Description ^†^	Total No. Animals (No. Males/No. Females) *	Treatment Route, Frequency	Treatment Timing and Duration	Challenge
1	Vehicle	7 (4 M/3 F)	30 min IV infusion, once daily	Days 4–15 PI: Vehicle	IM EBOV-exposed, 1000 pfu
2	5/2.5 mg/kg RDV	8 (4 M/4 F)	30 min IV infusion, once daily	Day 4 PI: 5 mg/kg RDV Days 5–15 PI: 2.5 mg/kg RDV	
3	10/5 mg/kg RDV	8 (4 M/4 F)	30 min IV infusion, once daily	Day 4 PI: 10 mg/kg RDV Days 5–15 PI: 5 mg/kg RDV	
4	20/10 mg/kg RDV	7 (3 M/4 F)	30 min IV infusion, once daily	Day 4 PI: 20 mg/kg RDV Days 5–15 PI: 10 mg/kg RDV	

^†^ RDV treatment was administered as a single loading dose on the first day of treatment, followed by once-daily maintenance doses (x/y mg/kg). * The number of animals reflects the number of animals completing the in-life phase. Two animals were removed from the study (1 each from Groups 1 and 4). F, female; M, male.

**Table 2:** Schedule of study events.

Procedure	Days Pre-Inoculation							Days Post-Inoculation																					
	−8	−7	−6 GLP Start	−5	−4	−3, −2, −1	0 ^a^ Virus Exposure	1	2	3	4	5	6	7	8	9	10	11	12	13	14	15	16	17–20	21	22–27	28	29–41	42 or T ^b^
Move to ABSL-4	X																												
Health Status Check ^c^												X	X	X	X	X	X	X	X	X	X	X	X	X	X				
Continuous Telemetry Monitoring				X	X	X	X	X	X	X	X	X	X	X	X	X	X	X	X	X	X	X	X	X	X	X	X	X	X
Acclimation	X	X	X	X	X	X																							
EBOV Exposure							X																						
RDV Treatment ^d^											X	X	X	X	X	X	X	X	X	X	X	X							
AM Observation (0600–1000)	X ^e^	X	X	X	X ^e^	X	X	X	X	X	X	X	X	X	X	X	X	X	X	X	X	X	X	X	X	X	X	X	X
AM Physical (Anesthetized)							X																X		X		X		X
Viral RNA (RT-PCR)							X				X	X	X	X		X		X			X		X		X		X		X
Hematology, Serum Chemistry, Coagulation							X				X		X	X		X		X			X				X		X		X
Virus Sequencing ^f^											X	X	X	X		X					X								
Viremia (Plaque Assay)							X							X		X									X		X		X
Bioanalysis (PK) Plasma ^g^											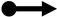						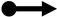												

^a^ Samples on Day 0 were collected prior to virus exposure. ^b^ Terminal samples were not collected on animals euthanized on a day on which a scheduled blood collection event had already occurred. ^c^ Additional observations when the responsiveness score was ≥ 1 (clinical signs of illness are apparent) for at least 1 animal; observations occurred every 3–7 h. ^d^ A loading dose was administered on Day 4 PI and maintenance doses on Days 5–15 PI. ^e^ On Days −8 and −4, awake observations were performed in the afternoon. ^f^ Virus sequence analysis was conducted on samples found to be PCR positive for viral RNA. ^g^ The bioanalysis plasma sampling schedule is represented schematically by arrows. ABSL-4, animal biosafety level 4; PK, pharmacokinetic; RT-PCR, reverse-transcription polymerase chain reaction; T, terminal.

**Table 3:** Summary of the survival of EBOV/Kikwit-infected rhesus monkeys treated with a once daily administration of remdesivir beginning 4 days after inoculation.

	Group 1 Vehicle (N = 7)	Group 2 RDV 5/2.5 mg/kg (N = 8)	Group 3 RDV 10/5 mg/kg (N = 8)	Group 4 RDV 20/10 mg/kg (N = 7)
Survival to Day 42, N (%)	1 (14.3%)	6 (75%)	7 (87.5%)	5 (71.4%)
*p*-value versus vehicle group *	—	0.032	**0.009**	0.051

* *p*-values are from a one-sided Fisher’s exact test using a predefined stepdown approach for multiple comparisons as follows: Survival in Group 3 and Group 1 was compared at a one-sided significance level of 0.05. If the difference between Groups 3 and 1 was statistically significant, survival in Group 4 and Group 1 would be compared at a one-sided significance level of 0.05. If this difference was statistically significant, survival in Group 2 and Group 1 would be compared at a one-sided significance level of 0.05 (see Section 2.15). Statistically significant values are indicated in boldface font.

**Table 4:** Serum infectious virus (log_10_ pfu/mL).

Days PI	Parameter	Group 1 Vehicle	Group 2 RDV 5/2.5 mg/kg	Group 3 RDV 10/5 mg/kg	Group 4 RDV 20/10 mg/kg
0	Mean (range)	1.60 (1.60–1.60)	1.60 (1.60–1.60)	1.60 (1.60–1.60)	1.60 (1.60–1.60)
	n	7	8	8	7
6	Mean (range)	6.41 (5.87–6.95)	NA	NA	NA
	n	2	0	0	0
7	Mean (range)	4.67 (2.90–5.52)	3.34 (1.60–5.04)	2.37 (1.60–4.41)	2.68 (1.60–2.90)
	n	4	6	7	6
8	Mean (range)	5.80 (NA)	4.74 (NA)	1.60 (NA)	NA
	n	1	1	1	0
9	Mean (range)	3.64 (2.90–5.13)	2.38 (1.60–2.90)	1.97 (1.60–2.90)	1.79 (1.60–2.90)
	n	3	5	7	7
10	Mean (range)	NA	NA	NA	2.90 (NA)
	n	0	0	0	1
12	Mean (range)	NA	NA	NA	2.90 (NA)
	n	0	0	0	1
21	Mean (range)	1.60 (NA)	1.60 (1.60–1.60)	1.60 (1.60–1.60)	1.60 (1.60–1.60)
	n	1	6	7	5
28	Mean (range)	1.60 (NA)	1.60 (1.60–1.60)	1.60 (1.60–1.60)	1.60 (1.60–1.60)
	n	1	6	7	5
42	Mean (range)	1.60 (NA)	1.60 (1.60–1.60)	1.60 (1.60–1.60)	1.60 (1.60–1.60)
	n	1	6	7	5

Samples in which infectious virus was detected at levels <LLOQ were assigned a value of 2.90 log_10_ pfu/mL, and samples in which infectious virus was not detected (<LOD) were assigned a value of 1.60 log_10_ pfu/mL. NA, not applicable.

**Table 5:** Mean pharmacokinetic parameters for RDV, GS-704277, and GS-441524 on Days 4 and 10 PI.

Group	Dose mg/kg	Analyte	C_max_ ng/mL		AUC_0–24_ h·ng/mL	
			Day 4 PI	Day 10 PI	Day 4 PI	Day 10 PI
2	5/2.5	RDV	2280	1670	1280	1110
3	10/5	RDV	3150	2650	1770	1540
4	20/10	RDV	6900	5600	4040	3660
2	5/2.5	GS-441524	93.5	68.5	807	616
3	10/5	GS-441524	193	153	1500	1410
4	20/10	GS-441524	493	662	4050	10,200
2	5/2.5	GS-704277	263	236	475	595
3	10/5	GS-704277	601	416	909	1050
4	20/10	GS-704277	1720	2060	2940	7680

C_max_, maximum observed concentration.

**Table 6:** Summary of notable pathology findings.

Nonsurvivors			Survivors		
**Gross Changes**	Liver (pale, friable, and/or enlarged)	91% (10 of 11)	**Gross Changes**	Lymph nodes (enlarged)	89% (17 of 19)
	Skin (rash)	82% (9 of 11)		Spleen (firm and/or enlarged)	68% (13 of 19)
	Spleen (firm and/or enlarged)	82% (9 of 11)		Pulmonary adhesions	21% (4 of 19)
	GI mucosa (hemorrhage)	73% (8 of 11)	**Microscopic Changes**	Spleen and/or lymph nodes (lymphoid hyperplasia of the white pulp)	100% (19 of 19)
	Testes (hemorrhage)	67% (4 of 6)			
	Lung (non-collapsing)	45% (5 of 11)		Liver (inflammation)	89% (17 of 19)
	Kidneys (pale)	36% (4 of 11)		IHC+ and ISH+ in 1 immune-privileged site (eye or testes) ^b^	16% (3 of 19)
**Microscopic Changes**	IHC+/ISH+, multiple tissues ^a^	100% (11 of 11)			
	Spleen (lymphoid depletion)	100% (11 of 11)		IHC+ in 1–2 non-immune-privileged tissues ^c^	16% (3 of 19)
	Lung (inflammation)	100% (11 of 11)			
	Lymph node (lymphoid depletion and lymphocytolysis)	91% (10 of 11)			
	Liver (degeneration and necrosis with inflammation)	91% (10 of 11)			
	Testes (hemorrhage)	83% (5 of 6)			
	Kidney (tubular epithelial degeneration and necrosis)	64% (7 of 11)			
	Muscle at challenge site (inflammation and degeneration)	55% (6 of 11)			
	Duodenum (necrosis and hemorrhage)	55% (6 of 11)			

^a^ Positive IHC and ISH staining was identified in all examined tissue for Groups 1–3 with few exceptions. Group 4 animals that died had reduced signal for most tissues and were negative for some tissues. ^b^ Eye (2 animals) and testes (1 animal). ^c^ Lung and heart (1 animal), kidney (1 animal), and corpus striatum (1 animal). The intensity of color corresponds to the prevalence of the characteristic.

## Data Availability

The data presented in this study are available upon request from the corresponding author and with permission from Gilead Sciences and JPEO-CBRND.

## Supplementary Materials

The following supporting information can be downloaded at: https://www.mdpi.com/article/10.3390/v16121934/s1, Figure S1: Representative Histopathology of a Vehicle-Treated Nonsurvivor and an RDV-Treated Survivor; Table S1: Summary of Survival, Viral RNA, and Notable Clinical and Telemetry Observations.

## Disclaimers

The opinions, interpretations, conclusions, and recommendations presented are those of the authors and are not necessarily endorsed by the U.S. Army, Department of Defense, or the U.S. Food and Drug Administration. The funders had no role in the design of the study; in the collection, analyses, or interpretation of data; in the writing of the manuscript; or in the decision to publish the results. The use of trade or manufacturers’ names in this publication does not constitute an official endorsement of any commercial products. This report may not be cited for purposes of advertisement. Contractor affiliation of some authors does not imply endorsement by the U.S. Government of any contractor.
