# Supplementary material for: A Randomized, Blinded, Vehicle-Controlled Dose-Ranging Study to Evaluate and Characterize Remdesivir Efficacy Against Ebola Virus in Rhesus Macaques
Source: Viruses. 2024 Dec 18;16(12):1934. doi: 10.3390/v16121934 (PMC11680158; doi:10.3390/v16121934)
Supplement: Supplementary file 1 [file viruses-16-01934-s001.zip › viruses-3366219-supplementary.pdf]

Supplemental Material

Table S1. Summary of Survival, Viral RNA, and Notable Clinical and Telemetry Observations.

Group	Subject ID	Survival (Day PI)[a]	RT-PCR at Day 4 PI (log_{10} ge/mL)	Peak RT-PCR Value (log_{10} ge/mL)	Peak RT-PCR (Day PI)	RT-PCR Neg[b] (Day PI)	Onset of Signif. Body Temp. Increase (Day)	Onset of Fever (Day)	Last Fever Day[c]	Hyper-pyrexia Present (Y/N)	Onset of Score[d] (Day PI)	Max Resp. Score	Rash Onset (Day PI)	Facial Swell. Onset (Day PI)	Bleed. Onset (Day PI)	Motor Dysfnct. Onset (Day PI)
Group 1 Vehicle	1	11	5.38	9.15	7	NA	3.65	4.27	NA	Y	7	3[e]	7	NA	7	NA
	2	42	5.99	7.60	6	16	3.94	3.94	13.44	Y	7	2	7	NA	11	NA
	3	9	7.18	8.45	6	NA	3.23	3.33	NA	Y	6	4	5	NA	7	NA
	4	7	8.67	10.30	6	NA	3.31	3.40	NA	N	5	4	5	NA	NA	6
	5	8	7.92	9.04	6	NA	3.23	3.29	NA	N	5	4	5	NA	NA	7
	6	7	6.45	9.56	6	NA	3.10	3.31	NA	Y	5	4	5	NA	NA	NA
	7	7	7.97	9.49	5	NA	3.00	3.17	NA	Y	5	4	5	NA	NA	NA
Group 2 RDV 5/2.5 mg/kg	8	42	6.03	6.69	5	14	3.08	3.15	14.23	Y	7	2	8	NA	8	8
	9	9	6.15	8.72	7	NA	3.10	3.31	NA	Y	6	4	6	NA	9	9
	10	42	6.09	7.91	6	14	3.56	4.37	25.02	N	9	2	9	NA	7	NA
	11	42	5.81	6.36	5	14	3.29	3.33	12.17	N	7	1	NA	NA	NA	NA
	12	42	8.65	8.65	6	21	3.23	3.25	22.35	Y	7	2	11	9	9	10
	13	42	6.53	8.29	5	14	3.25	3.37	20.58	Y	6	3	NA	10	9	11
	14	8	5.55	8.60	8	NA	3.71	3.92	NA	Y	6	4	6	NA	NA	NA
	15	42	7.92	8.78	6	16	3.25	3.35	16.23	Y	7	2	7	10	9	NA

Table S1. Cont'd.

Group	Subject ID	Survival (Day PI)[a]	RT-PCR at Day 4 PI ($\log_{10}$ ge/mL)	Peak RT-PCR Value ($\log_{10}$ ge/mL)	Peak RT-PCR (Day PI)	RT-PCR Neg[b] (Day PI)	Onset of Signif. Body Temp. Increase (Day)	Onset of Fever (Day)	Last Fever Day[c]	Hyper-pyrexia Present (Y/N)	Onset of Score[d] (Day PI)	Max Resp. Score	Rash Onset (Day PI)	Facial Swell. Onset (Day PI)	Bleed. Onset (Day PI)	Motor Dysfnct. Onset (Day PI)
Group 3 RDV 10/5 mg/kg	16	42	5.01	5.60	5	7	3.58[f]	3.94	9.56	N	6	2	6	NA	NA	NA
	17	42	5.29	6.78	6	14	3.54	3.62	12.50	Y	NA	0	NA	NA	NA	NA
	18	42	6.24	8.22	7	14	3.29	3.44	14.54	Y	6	3	6	12	7	12
	19	42	6.24	7.19	5	9	3.33	3.35	8.29	Y	7	2	7	NA	NA	NA
	20	42	6.84	6.84	4	11	2.58	2.60	9.60	N	NA	0	NA	NA	NA	NA
	21	42	5.88	7.49	5	11	3.37	3.60	14.62	Y	NA	0	NA	NA	10	NA
	22	8	8.64	9.47	6	NA	3.37	3.44	NA	Y	5	4	5	NA	NA	NA
	23	42	6.82	8.54	5	14	3.75	4.29	13.44	N	9	1	NA	NA	9	NA
Group 4 RDV 20/10 mg/kg	24	10	7.97	8.22	5	NA	3.17	3.27	NA	N	7	4	7	8	NA	NA
	25	42	6.00	6.38	5	9	3.29	3.35	9.19	N	9	1	NA	NA	NA	NA
	26	12	7.88	8.36	5	9	3.19	2.83	NA	Y	6	3[g]	7	7	11	10
	27	42	8.25	8.74	5	14	2.75	3.17	22.44	Y	6	3	7	8	11	12
	28	42	5.49	7.19	6	11	5.10	5.73	7.48	N	7	1	NA	NA	NA[h]	NA
	29	42	6.83	7.02	5	11	3.25	3.31	12.52	Y	NA	0	NA	NA	NA	NA
	30	42	6.49	8.10	5	11	3.44	3.46	15.56	Y	8	1	11	9	8	NA

[a] Animals surviving to Day 42 PI were deemed survivors; surviving animals were euthanized on Days 42 and 43 PI. [b] For surviving animals, the first day on which RNA was undetectable (below the limit of detection) by RT-PCR. [c] For surviving animals, the last day on which a significant, sustained fever was observed. [d] Onset of responsiveness score ≥ 1. [e] Found deceased. [f] This animal additionally had an unexplained period of significant temperature increase at 0.02 days. [g] Blood urea nitrogen and creatinine values prompted euthanasia. [h] Menstruation on Day 0. Yellow shaded rows denote nonsurvivors. NA, not applicable; RT-PCR, reverse-transcription polymerase chain reaction.

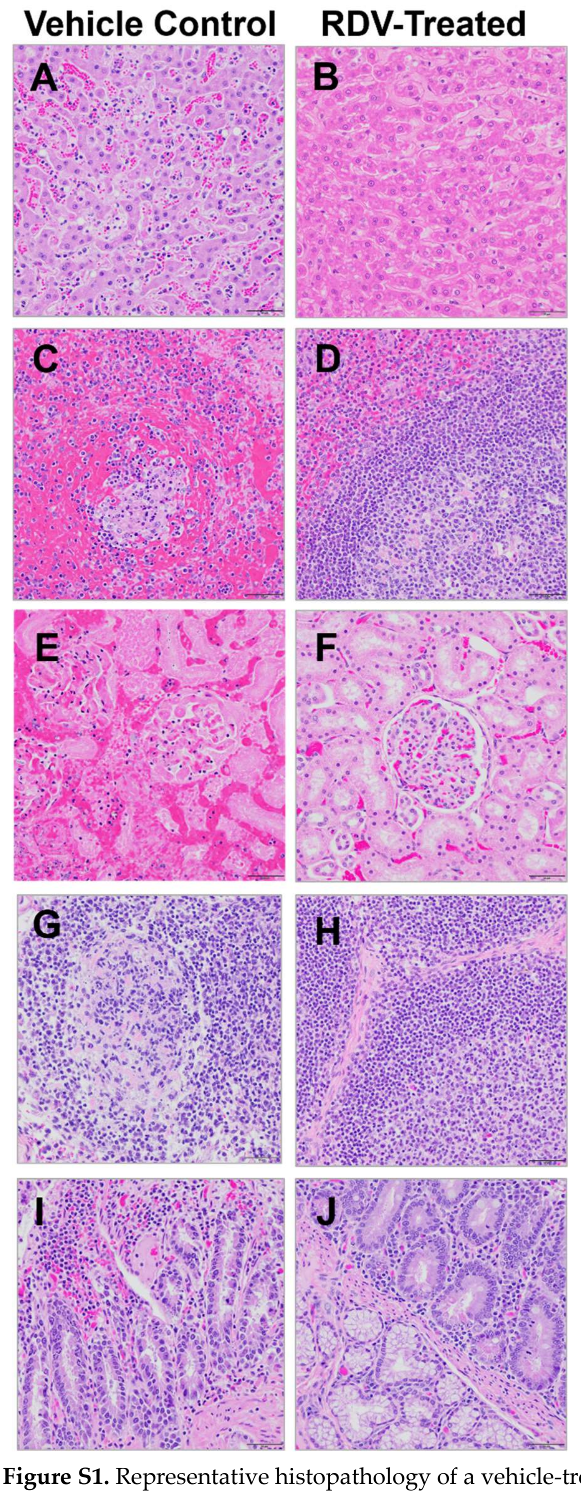

Figure S1. Representative histopathology of a vehicle-treated nonsurvivor and an RDV-treated survivor. Tissue sections of liver (A and B), spleen (C and D), kidney (E and F), inguinal lymph node (G and H), and duodenum (I and J) from a vehicle-treated nonsurvivor (left panel) and an RDV-treated survivor (right panel) were stained by hematoxylin and eosin. In contrast to the RDV-treated survivor, tissues of the nonsurvivor exhibited degeneration and necrosis in the liver and kidney, lymphoid depletion in the spleen and lymph node, and hemorrhage and necrosis in the duodenum. Scale bar, 50 μm.
